# Supplementary figures and images for: Interferon signaling suppresses the unfolded protein response and induces cell death in hepatocytes accumulating hepatitis B surface antigen
Source: PLoS Pathog. 2021 May 12;17(5):e1009228. doi: 10.1371/journal.ppat.1009228 (PMC8143404; doi:10.1371/journal.ppat.1009228)

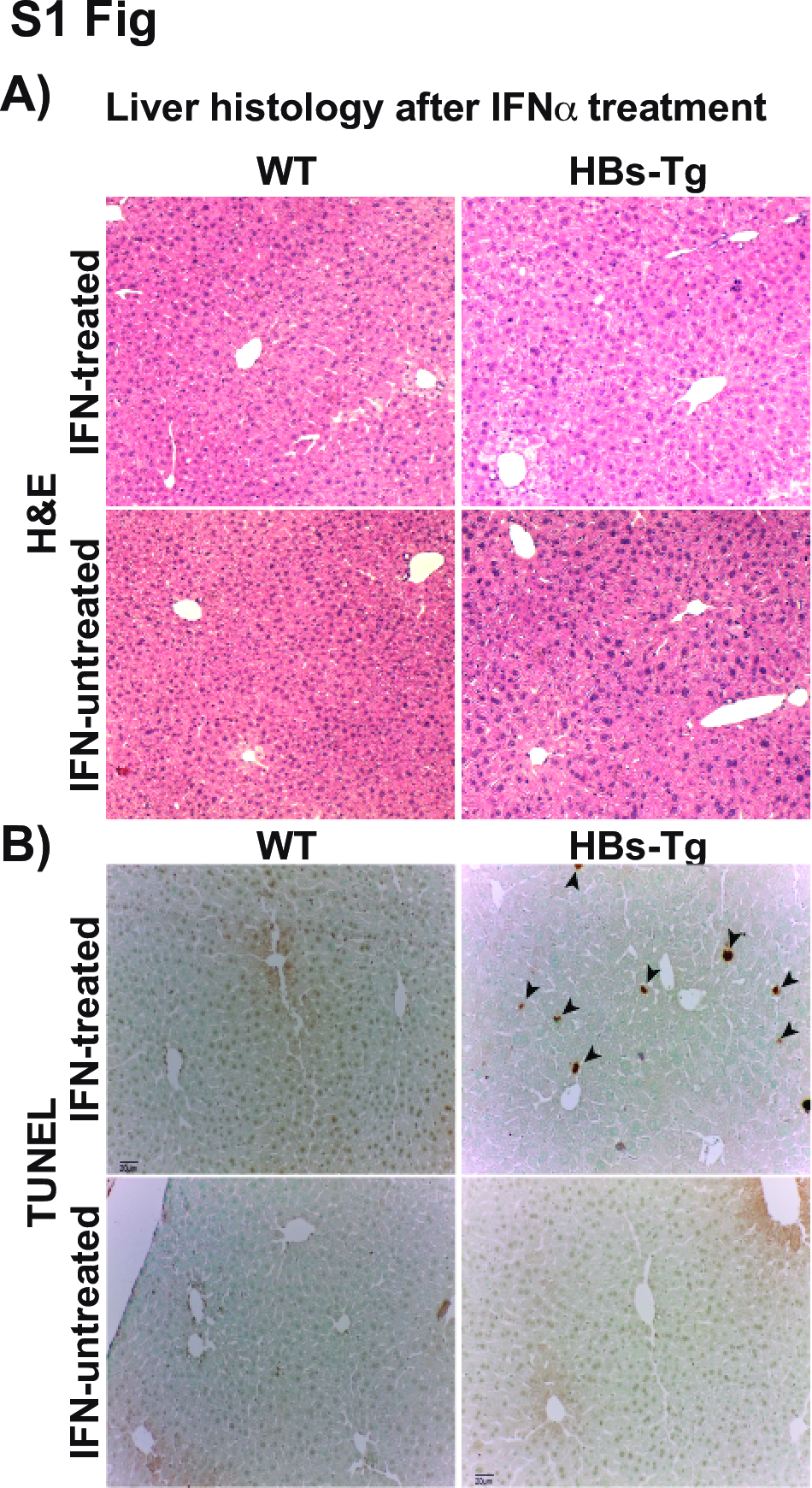

Supplement: S1 Fig — (A)The photomicrograph panels show representative Hematoxylin and Eosin staining at 24 hours after IFNα in WT (top left) and HBs-Tg mice (top right). Untreated controls are shown on the bottom panels, respectively. (B) The panels show representative TUNEL staining at 16 hours after IFNα treatment in WT mice (top left) and HBs-Tg mice (top right). The arrows show representative apoptotic cells in HBs-Tg mice. Untreated controls are shown on the bottom panels, respectively. (TIF) [file ppat.1009228.s001.tif]

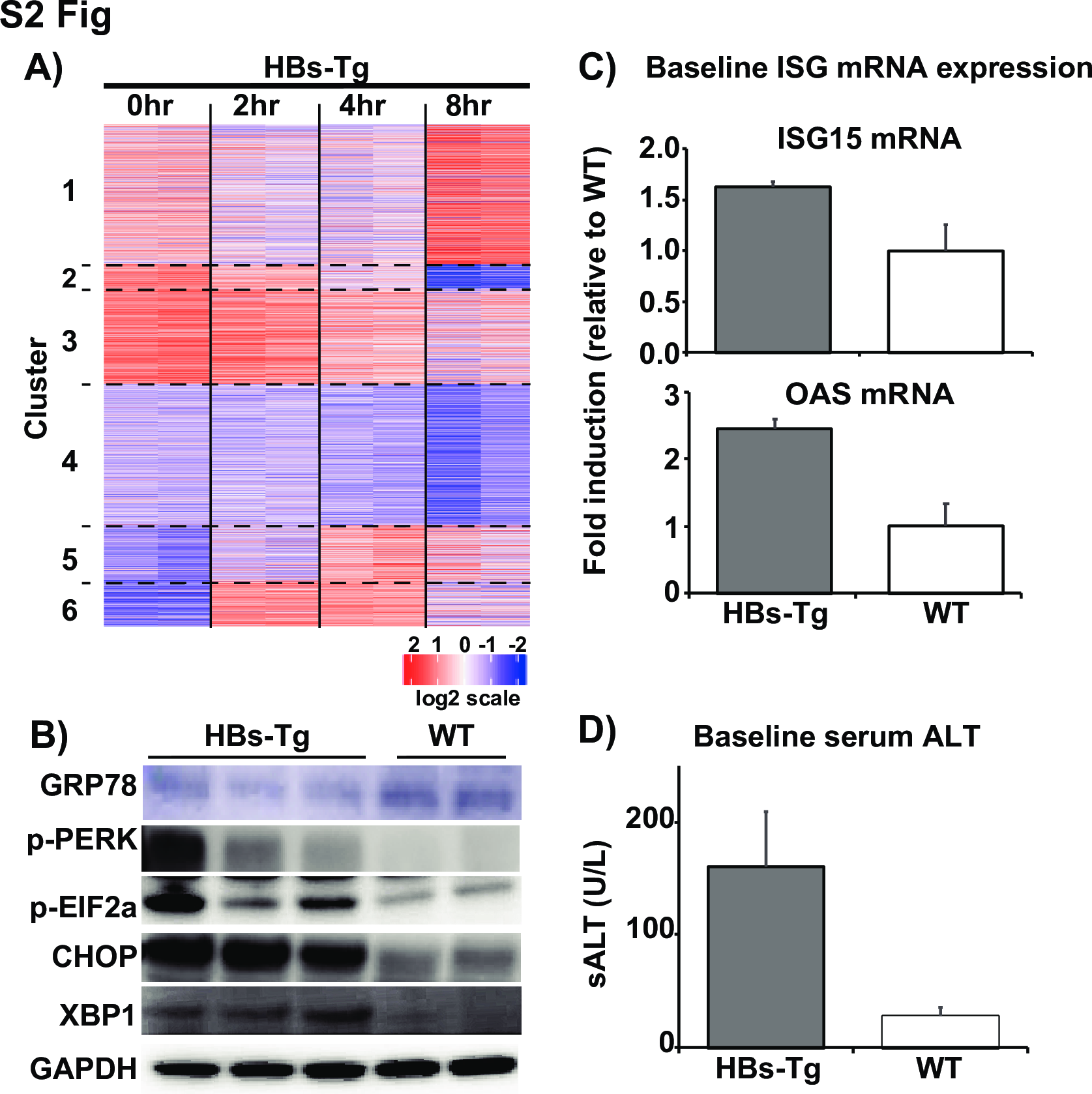

Supplement: S2 Fig — (A) The gene expression patterns used to group genes of similar expression kinetics into 6 distinct clusters. The heatmap shows the gene expression kinetics at 0, 2, 4, and 8-hours after IFN treatment in HBs-Tg mice. (B-D) Baseline expression of UPR markers, ISGs and serum ALT in non-treated 107-5D HBs-Tg mice. (B) The immunoblots show the expression of UPR molecules like CHOP, XBP1 and GRP78 in non-treated HBs-Tg mice compared with normal WT mice. (C) The graphs show the mRNA expression of ISG15 (top), and OAS (bottom) in non-treated HBs-Tg mice compared with normal WT mice. (D) Serum ALT levels in non-treated HBs-Tg mice compared with normal WT mice. (TIF) [file ppat.1009228.s002.tif]

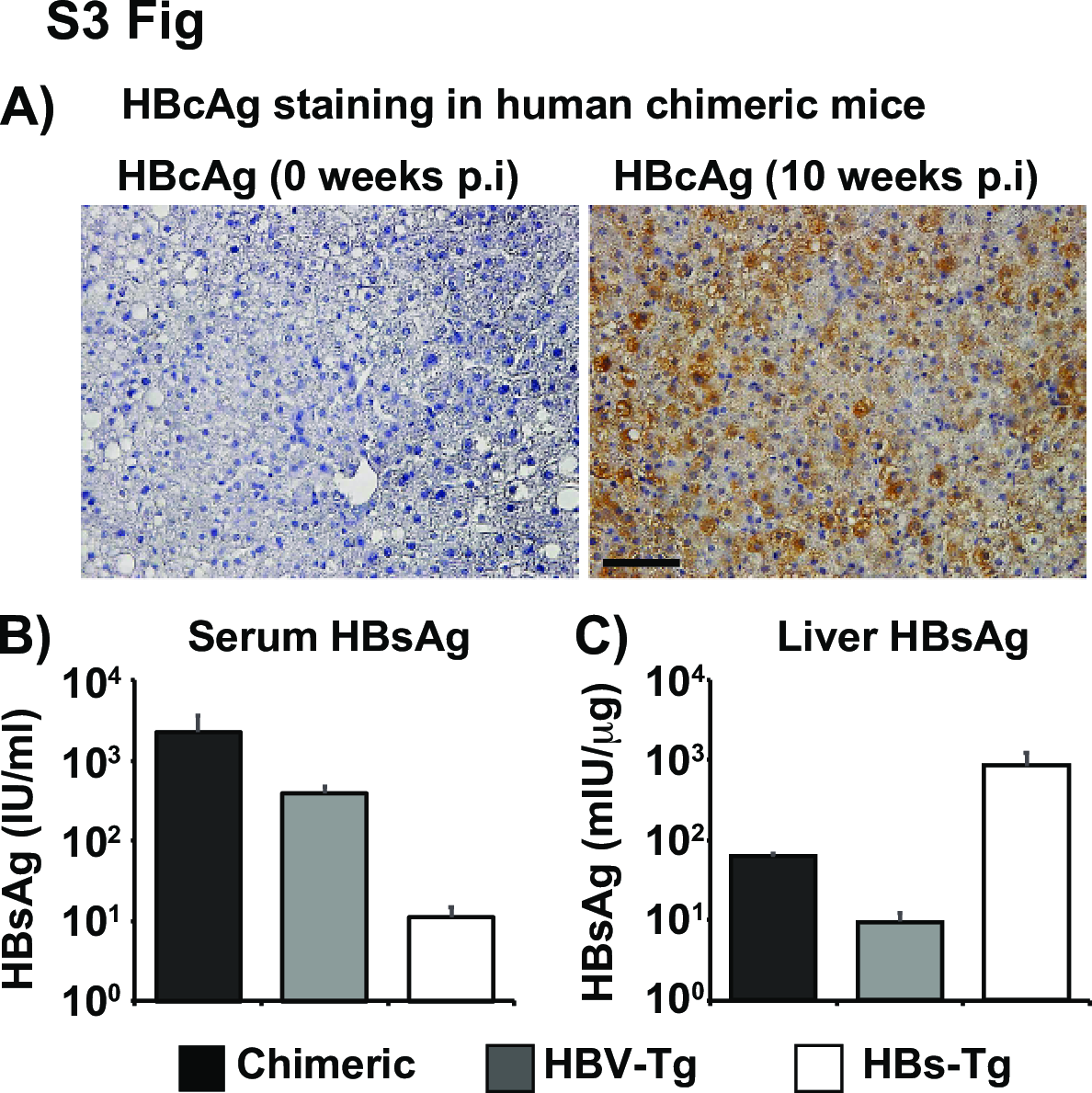

Supplement: S3 Fig — (A) The photomicrographs show HBV core antigen (HBcAg) staining in non-HBV infected (0 weeks) (left panel) and at 10 weeks after HBV inoculation (right panel) in humanized liver chimeric mice. (B-C) Characterization of the baseline extracellular and intracellular HBsAg levels in non-treated HBV infected chimeric, HBV-Tg and HBs-Tg mice. (B) A graph showing the serum HBsAg levels between chimeric mice, HBV-Tg and HBs-Tg mice. (C) A graph showing the intracellular HBsAg levels per μg of liver protein between chimeric mice, HBV-Tg and HBs-Tg mice. (TIF) [file ppat.1009228.s003.tif]

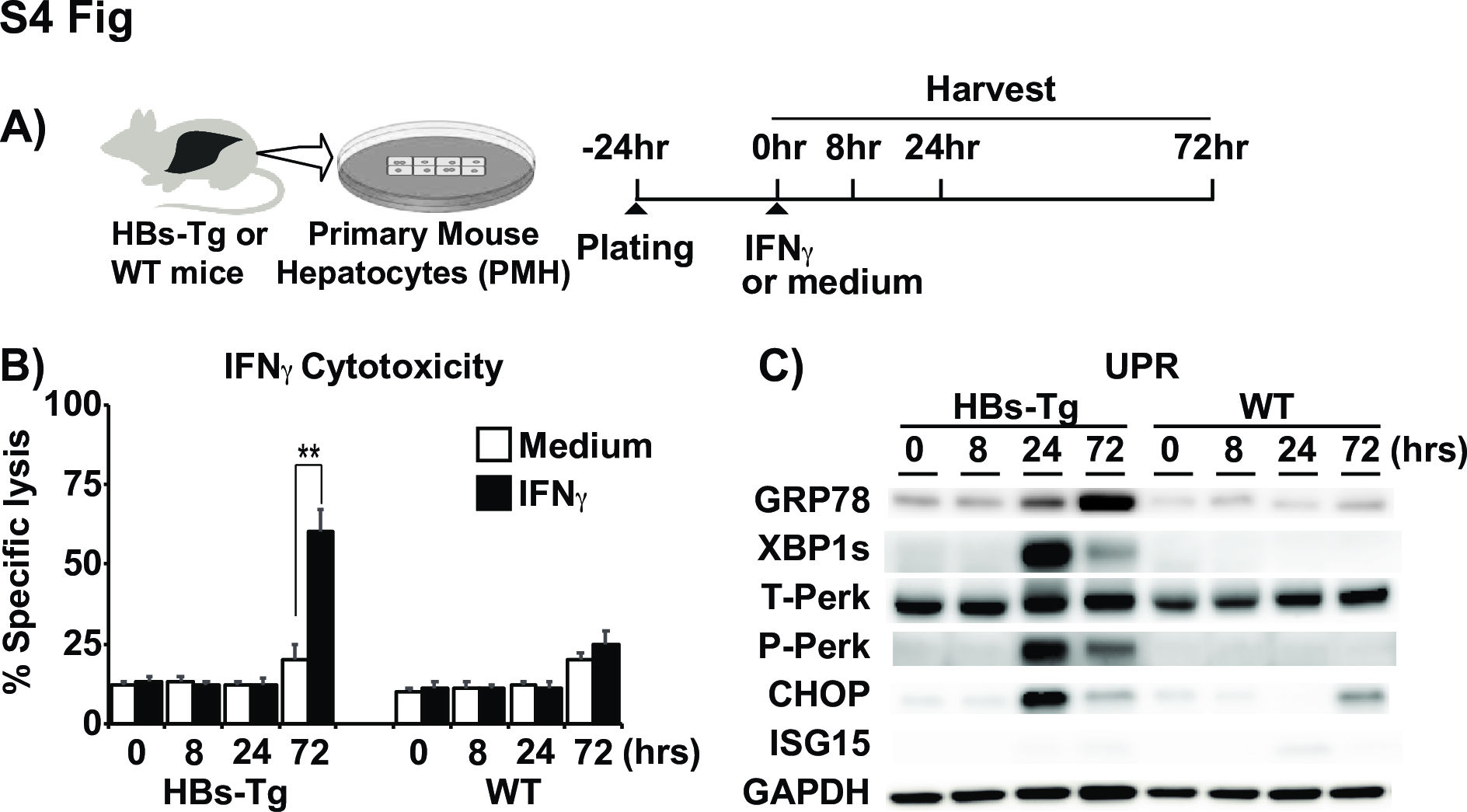

Supplement: S4 Fig — (A) Timeline showing how primary mouse hepatocytes (PMHs) from both HBs-Tg and WT mice were isolated, plated, and treated with either IFNγ, while monitoring cytotoxicity at the specified time points. (B) The graph shows significant LDH increase in HBs-Tg derived PMHs 72 hours after IFNγ addition. (C) The immunoblots show the effect of IFNγ treatment on UPR-related proteins at specified time points. (TIF) [file ppat.1009228.s004.tif]

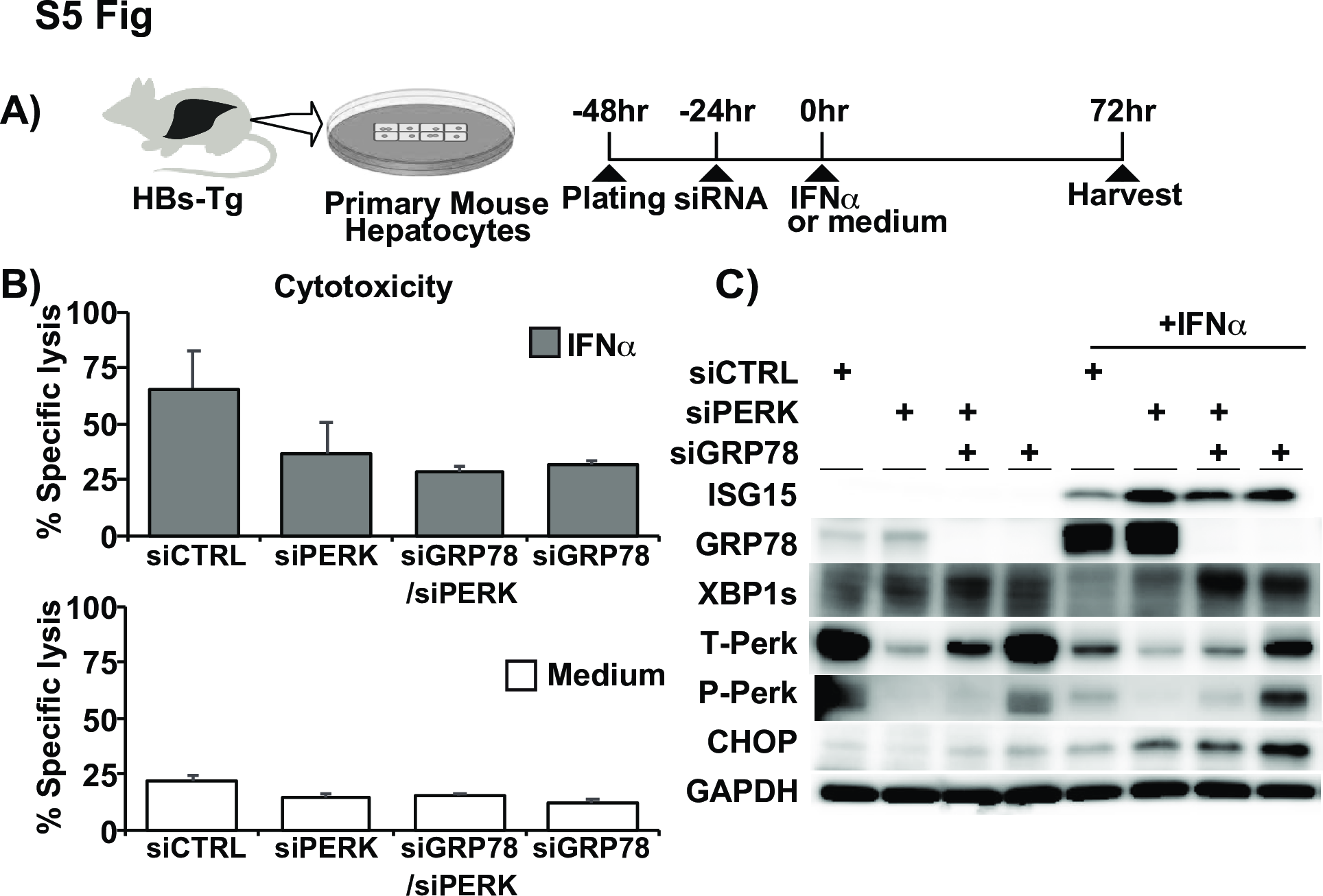

Supplement: S5 Fig — (A) Experimental design. Small interfering RNA (siRNA) targeting PERK, GRP78 or control scramble siRNA (siControl) (15 μM) were transfected to primary mouse hepatocytes (PMHs) from HBs-Tg mice before IFNα treatment. (B) LDH levels in the supernatant of cultured HBs-Tg PMHs after treatment with IFNα (top graph) or vehicle (medium) (bottom graph) following knockdown of each UPR-related molecule by specific siRNA. (C) Representative immunoblots showing UPR-related protein levels in the absence/presence of IFNα after indicated specific target downregulation by siRNA. (TIF) [file ppat.1009228.s005.tif]

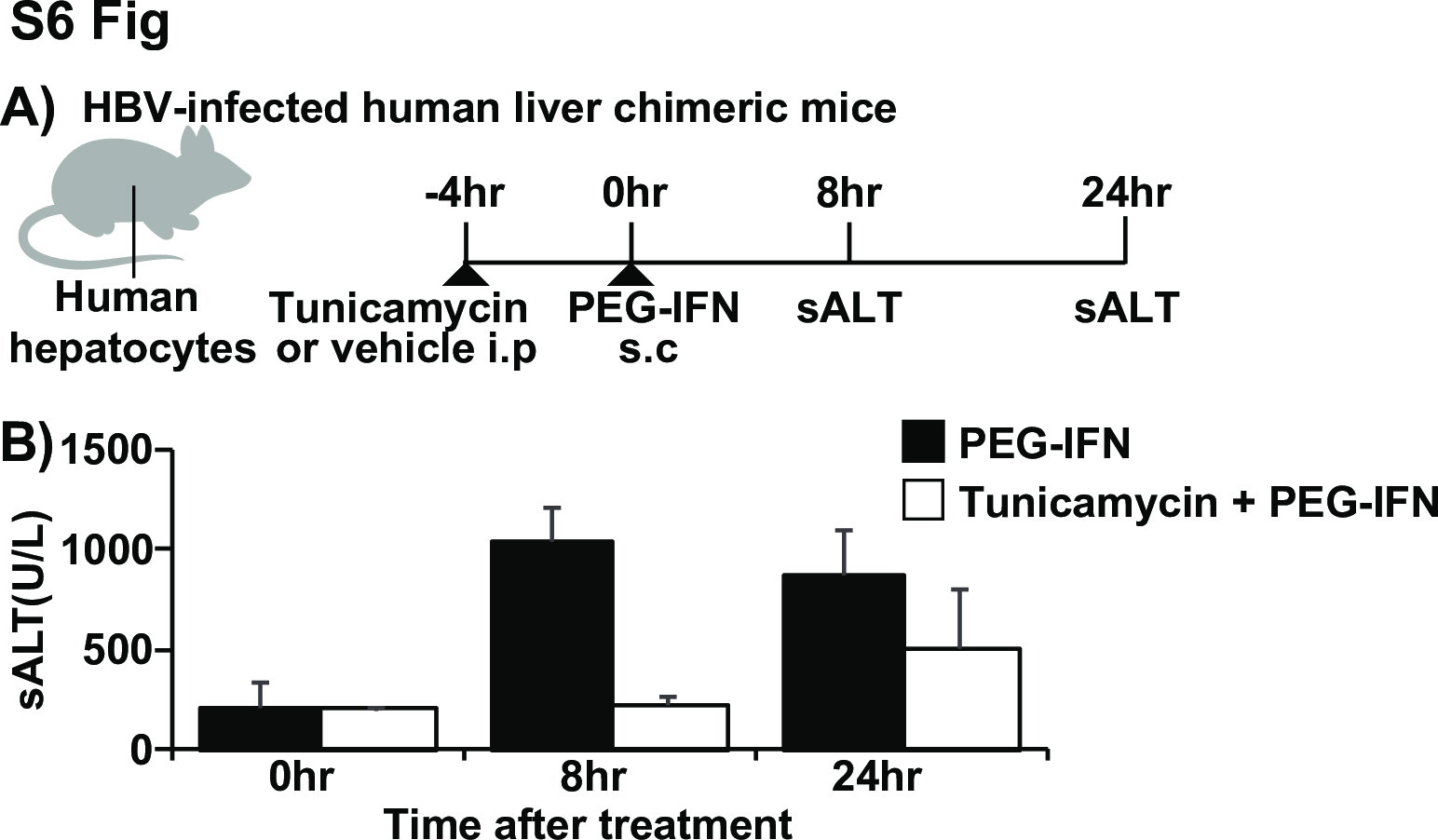

Supplement: S6 Fig — (A) Experimental design to test the effect of low dose tunicamycin administration (0.1mg/kg) on PEG-hIFN-induced liver injury in HBV infected chimeric mice. PEG-hIFN2α (25ng/g) or saline were intravenously injected to HBV infected chimeric mice at 4 hours after tunicamycin administration. (B) sALT levels at 8 and 24 hours after PEG-hIFN2α injection in tunicamycin (TUN) treated chimeric mice compared with controls. (TIF) [file ppat.1009228.s006.tif]
